# Playing Chemical Plant Environmental Protection Games with Historical Monitoring Data

**DOI:** 10.3390/ijerph14101155

**Published:** 2017-09-29

**Authors:** Zhengqiu Zhu, Bin Chen, Genserik Reniers, Laobing Zhang, Sihang Qiu, Xiaogang Qiu

**Affiliations:** 1 College of Information System and Management, National University of Defense Technology, Changsha 410073, China; admin@steven-zhu.me (Z.Z.); qiusihang@gmail.com (S.Q.); Michael.qiu@139.com (X.Q); 2 Faculty of Technology, Policy and Management, Safety and Security Science Group (S3G), Delft University of Technology (TU Delft), 2628 BX Delft, The Netherlands; g.l.l.m.e.reniers@tudelft.nl (G.R.); laobingzhang.nudt@gmail.com (L.Z.); 3 Faculty of Applied Economics, Antwerp Research Group on Safety and Security (ARGoSS), University Antwerpen, 2000 Antwerp, Belgium; 4 Centre for Economics and Corporate Sustainability (CEDON), KULeuven, Campus Brussels, 1000 Brussels, Belgium

**Keywords:** Chemical Plant Environmental Protection, Stackelberg Security Games, source estimation methods, historical monitoring data, game theory

## Abstract

The chemical industry is very important for the world economy and this industrial sector represents a substantial income source for developing countries. However, existing regulations on controlling atmospheric pollutants, and the enforcement of these regulations, often are insufficient in such countries. As a result, the deterioration of surrounding ecosystems and a quality decrease of the atmospheric environment can be observed. Previous works in this domain fail to generate executable and pragmatic solutions for inspection agencies due to practical challenges. In addressing these challenges, we introduce a so-called Chemical Plant Environment Protection Game (CPEP) to generate reasonable schedules of high-accuracy air quality monitoring stations (i.e., daily management plans) for inspection agencies. First, so-called Stackelberg Security Games (SSGs) in conjunction with source estimation methods are applied into this research. Second, high-accuracy air quality monitoring stations as well as gas sensor modules are modeled in the CPEP game. Third, simplified data analysis on the regularly discharging of chemical plants is utilized to construct the CPEP game. Finally, an illustrative case study is used to investigate the effectiveness of the CPEP game, and a realistic case study is conducted to illustrate how the models and algorithms being proposed in this paper, work in daily practice. Results show that playing a CPEP game can reduce operational costs of high-accuracy air quality monitoring stations. Moreover, evidence suggests that playing the game leads to more compliance from the chemical plants towards the inspection agencies. Therefore, the CPEP game is able to assist the environmental protection authorities in daily management work and reduce the potential risks of gaseous pollutants dispersion incidents.

## 1. Introduction

Controlling atmospheric pollution is essential for preserving today’s environment and there is a sense of urgency present due to the ever expanding chemical industrial activities. Indeed, byproducts generated during chemical production processes are noxious, even sometimes highly toxic, and often they are discharged to nearby atmospheric environments without purification treatment. In extreme cases, the atmospheric pollution incidents caused by spontaneous or anthropogenic activities can exert harmful or fatal effects on humans and natural environment [1]. As a result, the atmospheric quality in developing countries where the control on environmental pollution is absent or very low, is extremely poor [2], further leading to substantial health problems for the residents and to the potential destruction of the ecosystem. Recent results (e.g., [3,4]) imply that atmospheric pollution of chemical power plants can pose great health risks to surrounding occupants. Moreover, the importance of installing emission control devices for the power systems was highlighted. At present, a core issue of concern to those who manage the chemical cluster is the effective prevention and mitigation of impacts caused by risk accidents, and the implementation of effective management that can ensure safe production and social stability [5].

Faced with these problems, governments in developing countries have introduced a series of measures to abate atmospheric pollution [6,7]. For instance, chemical plants are required by law to dispose atmospheric pollutants through Purification Treatment Plants (PTPs) prior to releasing them into the air. However, there is evidence that chemical plants do not run PTPs in most instances, for economic reasons (e.g., profit maximization). It is often up to regulatory bodies or inspection agencies to enforce compliance by fining these chemical plants in the case that they violate pollution control regulations. However, on the one hand, many inspection agencies lack in inspecting resources; and, on the other hand, it is difficult for them to draw up an intelligent strategy to detect these irregularities. A simple solution would be used to forbid these factories to operate in the country. However, such a solution would perhaps solve the problem in a short term, but the downside is the devastating implications for the national economies. 

Previous work in addressing this non-compliance issue falls short of generating effective solutions for inspection agencies to optimize audit and detection practices. With the help of the government, the inspection agency is nonetheless equipped with atmospheric monitoring facilities to conduct air monitoring. However, without utilization of source estimation methods, it is still hard for inspection agencies to distinguish whether a factory violates or not. Besides, inspection agencies do not dispose of quantitative and effective methods to conduct their inspection schedules. Therefore, they have difficulties in dealing with this problem.

With recent developments and successful deployments in various domains, such as seaports, airports, airline flights and rapid transit systems [8,9], game-theoretic models are able to provide a rigorous and mathematically based method to quantitatively model the interaction between the inspection agency on the one hand and the chemical plants on the other. 

Game-theoretic models, especially Stackelberg Security Games (SSGs) are utilized in earlier studies to generate intelligent security strategies. A generic Stackelberg Game consists of two players [10], a leader (a defender) and a follower (an attacker), in which a defender attempts to optimally allocate her limited security resources to protect a set of targets against an adversary attempting to attack one of the targets to optimize his utility. In SSGs, the defender commits to a mixed strategy first while the follower can observe the mixed strategy and subsequently take an action to optimize his reward. A pure strategy of the defender is an assignment of her limited resources to a subset of targets, while a mixed strategy of the defender refers to a probability distribution over all possible pure strategies [11]. A marginal coverage vector over the targets is often used to represent mixed strategies of the defender (i.e., the coverage probability with which the defender will protect every target) [12]. The number of targets demanding protection and the defender’s coverage probability at target i can be denoted by N and $c_{i}$, respectively ($0\leq c_{i}\leq 1$, i=1…N). When the adversary attacks a target i, he will receive a reward $R_{i}^{a}$ if the target is not protected by the defender’s resource; otherwise, he will receive a penalty $P_{i}^{a}$. Conversely, the defender will get a penalty $P_{i}^{d}$ in the former case and a reward $R_{i}^{d}$ in the latter case. The expected payoff of the defender, $U_{i}^{d}$, and attacker, $U_{i}^{a}$, are computed as follows. 

(1) $$U_{i}^{d}=c_{i}\cdot R_{i}^{d}+(1-c_{i})\cdot P_{i}^{d}$$

(2) $$U_{i}^{a}=c_{i}\cdot P_{i}^{a}+(1-c_{i})\cdot R_{i}^{a},$$

Inspired by the success of applying defender-attacker SSGs for the protection of infrastructure including airports, ports and trains, such games have also been applied in the domains of chemical plant protection and environment protection with two orientations: Chemical Plant Protection Games (CPPs) and Green Stackelberg Games (GSGs). In the chemical security domain, a game-theoretic approach was utilized by Reniers et al. [13,14,15,16,17,18] to systematically study cooperation regarding safety and security investments within chemical clusters. Whether investing in safety and security by the stakeholders of plants, or not investing, is the main focus of their model. Then, Zhang and Reniers [19] introduced a simultaneous game-theoretic model called “CPP Game” to protect chemical plants from terrorist attacks, and subsequently they [20] extended their model to sequential games played by a leading defender and several types of following attackers. These initial successes pointed the way to major future applications in the CPP security domain, with major challenges in scaling up game-theoretic algorithms, to address bounded rationality of human adversaries and uncertainties in action execution and observation. Besides, GSGs also emerged up in recent years, applications of which mainly focused on protecting the environment, including forests, fish and wildlife [21]. One of the newer applications in this field was protecting forests [22], where spatial considerations are taken into enforcement decisions for the defender. Another area of interest was protecting endangered species, in which PAWS (Protection Assistant for Wildlife Security) [23] is a typical application. Additionally, an emerging application domain was that of ensuring the sustainability of fish resources [24,25]. In our work, the atmospheric pollutant prevention problem is different from the two domains of applying SSGs mentioned above. Our research goal is to protect the environment and reduce risks of gaseous pollutant dispersion accidents, while CPPs were developed to protect important properties and facilities from attacks. Moreover, GSGs, the concept of which is repeated SSGs, have not paid any attention to the issue of protecting atmospheric environment yet. The essence of GSGs is the models that are used to deal with adversaries who are characterized by bounded rationality. However, infraction data of discharging excessive atmospheric pollutants is absent in the past research. Therefore, our Chemical Plant Environment Protection Games (CPEPs), which are truly different from the general concept of GSGs, follow the way of basic SSGs.

In light of the above, we introduce a new game-theoretic model, which we called CPEP, since similar game-theoretic models have been successfully developed and applied in related domains. In the background of a chemical industrial park, chemical plants tend to maximize their profits by discharging excessive atmospheric pollutants without purification treatment, while inspection agencies are charged with the inspection of production processes of chemical plants. If an inspection agency observes irregularities within a chemical plant, it will be heavily fined. In this paper, CPEPs focus on generating an optimal daily management plan for the inspection agency against the interaction between the inspection agency and the chemical plants to reduce incidents’ risks and control air pollution.

The proposed CPEPs facilitate the decision-making process of the daily management work through the following contributions. Firstly, a novel game model named CPEPs in conjunction with source estimation methods is introduced to detect the irregularities of chemical plants. Secondly, two inspection resources including high-accuracy monitoring stations and gas sensor modules are modeled in CPEPs for the first time. Finally, CPEPs are built up based on historical monitoring data analysis. Therefore, the proposed method not only deals with atmospheric pollutants controlling problem, but also reduces the risks of gaseous pollutants’ incidents.

The remainder of this paper is organized as follows: Section 2 presents the main modeling process of CPEPs with corresponding baseline algorithms. Case studies are realized in Section 3 to illustrate how the models and algorithms proposed in this paper work. Finally, conclusions and real industrial practice are discussed in Section 5.

## 2. Model Description

In this section, CPEP model consisting of players, strategies, payoffs and solution concept is firstly built up in Section 2.1 and some definitions (e.g., pure strategy, mixed strategy, Strong Stackelberg Solution, Nash Equilibrium, etc.) will be given at the same time. Source estimation methods are briefly introduced in Section 2.1.3; interested readers are referred to Qiu and Zhu [26,27]. Then, baseline algorithms are introduced in Section 2.2 to deal with CPEPs. Finally, the combination work of applying game-theoretic model and source estimation methods is further clarified in Section 2.3. Table 1 lists key notations used in this paper.

### 2.1. CPEP Model

The envisioned game-theoretic model should provide an approach to deal with interactions between the intelligent adversaries, that is, the chemical plants (“attackers”) on the one hand and the inspection agency (“defenders”) on the other. The model should assist the inspection agency to carry out their audit and detection approach in a more efficient and effective way. Basically, if an inspection agency in a chemical industrial park is equipped with high-accuracy air quality monitoring stations and gas sensor modules, these inspection resources would be operated continuously (24/7), regardless of the cost, in present and past practice. Different from the present and past practice, we model this atmospheric pollution prevention problem as a defender-attacker Stackelberg Security Game.

Speaking in general terms, strategic players are included in a game-theoretic model. Thus, every player has a set of feasible actions or choices, which are called “pure strategies”. After these strategies are carried out or implemented, players will acquire a reward or receive a penalty correspondingly. Payoffs can be calculated accordingly (e.g., by Formulas (1) and (2)). Finally, solutions constituted of typical strategies are discussed in Section 2.1.4. The elements mentioned above are modeled step by step in CPEPs as explained hereafter.

#### 2.1.1. Players

In our research problem at hand, the defender is represented by the inspection agency and the attackers are represented by the chemical plants. The latter attempt to discharge excessive atmospheric pollutants to optimize their payoffs after observing the actions taken by the defender (we use “leader” or “defender” to refer to the inspection agency and “follower” or “attacker” to refer to the chemical plant in the remainder of this paper). The task of the defender is to optimize the operating schedules of high-accuracy air quality monitoring stations to achieve more compliance from the chemical plants, and at the same time, to reduce its own operational costs. Moreover, both the chemical plants and the inspection agency are assumed rational based on two basic reasons in this paper. First, both players in CPEPs are able to perceive their situation and the opposite player’s actions accurately. Second, the players tend to maximize their payoffs through intelligently planning their strategies. Meanwhile, the interactions between the inspection agency and the chemical plants are characterized by the following considerations: (i) Knowledge about the capabilities and locations of the high-accuracy air quality monitoring stations and gas sensor modules is available to the chemical plants, primarily from the long-term observation of these facilities; (ii) Basic knowledge about the chemical plants, for instance, the locations, main productions, byproducts, etc. is available to the inspection agency, since this information needs to be provided by the chemical plants; (iii) Knowledge about pure strategies of players is available to both parties.

In this article, we use Θ to represent the inspection agency and Ψ to refer to a chemical plant.

#### 2.1.2. Strategies

The pure strategy (i.e., a single management measure) of players within the context of a chemical industrial park is a binary choice (i.e., for the inspection agency, open the monitoring stations or close the monitoring stations; for the chemical plants, release the excessive atmospheric pollutants or not) in different time slices in one day. One day is assumed to be equally divided into T time slices and the defender is assumed to have R monitoring stations (i.e., R high-accuracy inspection resources). However, in practice, though the defender might have multiple monitoring stations, she operates these resources on the same states (e.g., in one time slice, turn on or turn off all the stations). Therefore, these inspection resources can be considered ”one resource”. A more in depth explanation of this operation is given in the case study. Besides, we use $S_{\Theta }$ and $S_{\Psi }$ to denote an index set of pure strategies for the inspection agency and the chemical plants respectively. Thus, the pure strategy set of the inspection agency can be denoted as $\sum_{\Theta }=\{\theta_{1},\ldots ,\theta |S_{\Theta }|\}$, while $\sum_{\Psi }=\{\psi_{1},\ldots ,\psi |S_{\Psi }|\}$ is the pure strategies set for the attacker. The formulated representations of $\theta_{i}$, $\psi_{i}$, $\left|S_{\Theta }\right|$ and $\left|S_{\Psi }\right|$ are shown in the following formulas:

(3) $$\theta_{i}=\Pi_{r\in |R|,t\in |T}\;s_{d}(r,t),$$

(4) $$\psi_{i}=\Pi_{t\in |T|}\;s_{a}(t),$$

(5) $$|S_{\Theta }|=2^{R\cdot T},$$

(6) $$|S_{\Psi }|=2^{T},$$

where $\theta_{i}$ represents a pure strategy for the defender while $\psi_{i}$ denotes a pure strategy for the attacker; the notation of |R| denotes |R|={1,2,…,R}; similarly, the parameter of |T| means |T|={1,2,…,T}; the notation of $s_{d}(r,t)$ means $s_{d}(r,t)\in \{open,close\}$ while the notation of $s_{a}(t)$ means $s_{a}(t)\in \{release,no\;release\}$; the cross product is denoted through Π; and the number of pure strategies for the inspection agency and the chemical plant is denoted through $\left|S_{\Theta }\right|$ and $\left|S_{\Psi }\right|$ and respectively.

According to Formula (3), a pure strategy of the defender is defined as a combination of operation states of monitoring stations in all time slices in a day. Similarly, a pure strategy of the attacker is defined as a combination of discharging states in all time slices in a day according to Formula (4). For instance, if time slices T in one day are set at two and the value of R is set at one, the pure strategies for both players in one day are shown in Table 2. At the same time, a mixed strategy refers to a probability distribution over all possible pure strategies. For the defender, we use $x_{i}\in [0,1]$ to indicate the probability of the defender utilizing the pure strategy $\theta_{i}\in \sum_{\Theta }$. In contrast, the chemical plant takes action after observing the inspection agency’s mixed strategy and he will choose the best strategy to respond rather than mixing his strategy, to this end, $q_{i}\in \{0,1\}$ is used to indicate the probability of the attacker utilizing the pure strategy $\psi_{i}\in \sum_{\Psi }$.

**Figure 1:**
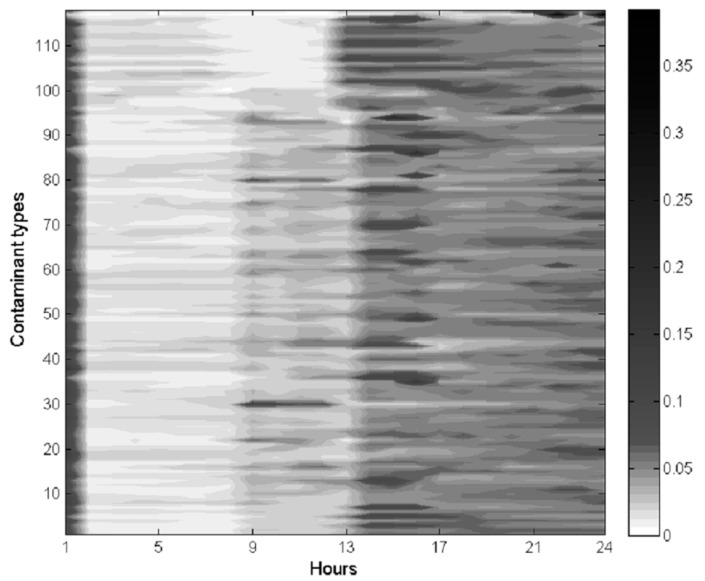
Daily hour-average concentration trend during the past year.

The division of one day determines how many pure strategies that the inspection agency and the chemical plants will have. A method based on historical discharging data is proposed in this paper to divide one day. Figure 1 illustrates a daily hour-average concentration trend detected by high-accuracy air quality monitoring stations during the past year. In the figure, the *X*-axis is the time series of one day while the *Y*-axis represents the main atmospheric contaminants monitored by monitoring stations. There are about 118 types of main atmospheric pollutants (e.g., Nitrogen oxides, Carbon oxides, VOCs, etc.) studied in this paper. The background color of this figure is white, which means concentration value of atmospheric pollutants is zero. Furthermore, a darker area represents higher gas concentrations. It can be concluded from the color-bar that black is darker than grey and white means that the concentration of the former is greater than that of the latter. In the figure, it is obvious that discharging behavior of chemical plants clearly has time characteristics. The discharging amount of atmospheric pollutants in the time unit of 12–24 h is far greater than that in the time unit of 1–12 h. Basically, production processes within chemical plants last for several hours. Hence, it is impractical to divide the time segment narrowly. Moreover, high-accuracy air quality monitoring stations are unsuitable to open and close frequently because a high start-up frequency may damage the facilities [28]. Therefore, it is reasonable to divide one day into two time slices in this paper.

This paper only offers a choice for readers to apply historical data into the modeling process. Interested readers can employ other reasonable approaches when determining the value of time slices in one day. Since the number of pure strategies is exponential to the value of T, a narrow division of one day will lead to high computation challenges. To simplify the modeling process and to ensure the facility safety, the value of T has an upper bound in most instances.

#### 2.1.3. Payoffs

In this section, source estimation methods are modeled into CPEPs to predict the infraction behavior of the chemical plants. The ability of source estimation methods successfully predicting the irregularities of chemical plants with only the discharging data from the gas sensor modules is defined as $\gamma_{1}$, while with the discharging data from the integrated information of monitoring stations combined with the gas sensor modules is defined as $\gamma_{2}$. The probability of $\gamma_{2}$ is assumed to be larger than that of $\gamma_{1}$ because monitoring data collected by high-accuracy air quality monitoring stations is more helpful in predicting the potential releasing spots. The source consists of two indicators: one is the location of the releasing spot and the other is the releasing rate of the discharging spot. After the potential releasing spots are calculated through source estimation methods when real-time monitoring data are applied as inputs, the inspection agency will send a law enforcement team to verify the infraction behavior.

The parameters explained hereafter are also determined to calculate the payoff of both defender and attacker from the point of view of the inspection agency. There are N chemical plants in a chemical industrial park and several high-accuracy air quality monitoring stations conducting surveillance. Other than high-accuracy air quality monitoring stations, an inspection agency is also assumed to have deployed a large number of portable gas sensor modules spread all over the chemical industrial park. Therefore, the inspection agency has a certain possibility to distinguish whether a factory is discharging atmospheric pollutants in the circumstance even if high-accuracy air quality monitoring stations are shut down. The operation cost of high-accuracy air quality monitoring stations in a time unit for the inspection agency is defined as $C_{d}$ while the operation of a purification treatment plant for treating atmospheric pollutants in a time unit for a chemical plant is $C_{a}$. Commonly, the operation cost of PTPs is much higher than that of high-accuracy air quality monitoring stations. If the $l^{th}$ chemical plant discharges atmospheric pollutants and the inspection agency fails to detect the infraction behavior, the chemical plant obtains a reward $R_{l}^{a}$ while the inspection agency gets a penalty $P_{l}^{d}$; conversely, if the inspection agency successfully detects the infraction behavior, the chemical plant receives a penalty $P_{l}^{a}$ while the inspection agency achieves a reward $R_{l}^{d}$. In developing countries (e.g., China and India), the government has published detailed regulations that if a chemical plant is caught of discharging excessive pollutants, it will be fined heavily. Part of the fine will be served as a reward for the work of the inspection agency. Thereby, it is assumed that $0\leq -P_{l}^{d}\leq R_{l}^{d}$ and $0\leq R_{l}^{a}\leq -P_{l}^{a}$. Primarily, the reward $R_{l}^{a}$ comes from discharging excessive atmospheric pollutants without purification treatment while the penalty $P_{l}^{d}$ comes from the pressure of public opinion and authorities. In addition, both the penalty $P_{l}^{a}$ and the reward $R_{l}^{d}$ come from forfeit.

The binary choice for the inspection agency (e.g., only one inspection resource is considered) and the chemical plant in one time slice constructs a payoff matrix, where the chemical plant is the row player while the inspection agency is the column player. Thus payoff tuples can be represented as $\left(u_{a},u_{d}\right)$ in Table 3. The payoff matrix can also be considered as payoffs in the circumstance of pure strategy for the inspection agency and the chemical plant when the value of T is set at one.

In the first case, when the high-accuracy air quality monitoring stations are open and the chemical plant is releasing excessive atmospheric pollutants, the payoff for the inspection agency is computed as the reward of a successful detection by high-accuracy air quality monitoring stations and gas sensor modules plus the penalty of unsuccessful detection by the inspection minus the operational costs of the high-accuracy air quality monitoring stations through the formula $\gamma_{2}\cdot R_{l}^{d}+(1-\gamma_{2})\cdot P_{l}^{d}-C_{d}$. Similarly, the difference in the second circumstance is the shutting down of the high-accuracy air quality monitoring stations compared to the first case, and thus the corresponding payoff for the inspection agency is calculated by the reward of successful detection by gas sensor modules plus the penalty of unsuccessful detection through the formula $\gamma_{1}\cdot R_{l}^{d}+(1-\gamma_{1})\cdot P_{l}^{d}$. The payoffs for the inspection agency are quite easy in the third and fourth cases, denoted as $C_{d}$ and 0 respectively. Analogously, in the first circumstance, the payoff for the chemical plant is computed as the reward of successfully discharging excessive atmospheric pollutants plus the penalty of unsuccessful infraction under the probability $\gamma_{2}$ through the formula $(1-\gamma_{2})\cdot R_{l}^{a}+\gamma_{2}\cdot P_{l}^{a}$. The difference for the chemical plant to compute his payoff in the second case is the probability, denoted as $\gamma_{1}$, compared to the first circumstance. The payoffs for the chemical plant are simple in the third and fourth cases, both denoted as $C_{a}$.

Then, the parameters $p_{a1}$, $p_{a2}$, $p_{a3}$ and $p_{a4}$ are used to represent the payoffs for the chemical plant under the pure strategy tuple of (release,open), (release,close), (no release,open) and (no release,close) respectively; similarly, the parameters $p_{d1}$, $p_{d2}$, $p_{d3}$ and $p_{d4}$ are used to represent the payoffs for the inspection agency under the pure strategy tuples mentioned above. Based on these parameters, the payoffs for both players under a pure strategy tuple of (θi,ψj) in T time slices are exhibited in the following formulas:

(7) $$u_{d}^{l}(\theta_{i},\psi_{j})=\sum_{k=1}^{4}N_{k}\cdot p_{dk},$$

(8) $$u_{a}^{l}(\theta_{i},\psi_{j})=\sum_{k=1}^{4}N_{k}\cdot p_{ak},$$

(9) $$\sum_{k=1}^{4}N_{k}=2^{T\cdot (R+1)}\;\forall N_{k}\in [0,2^{T\cdot (R+1)}]\text{​​}and\;N_{k}\in Z,$$

where the notation of $N_{k}$ denotes the number of the $k^{th}$ pure strategy tuples (i.e., (release,open), (release,close), (no release,open) and (no release,close)) under the pure strategy tuple of (θi,ψj) in T time slices. 

Formulas (7) and (8) represent calculating the summation of each product, that is, the multiplication of the number of the $k^{th}$ pure strategy tuples with the corresponding payoff. 

Moreover, in view of the above formulas and Table 3, the payoffs for the inspection agency and the chemical plant in the circumstance of mixed strategy can be shown as follows:

(10) $$u_{d}^{l}(x,q)=\sum_{i\in S_{\Theta }}\sum_{j\in S_{\Psi }}u_{d}^{l}(\theta_{i},\psi_{j})\cdot x_{i}\cdot q_{j}^{l},$$

(11) $$u_{a}^{l}(x,q)=\sum_{i\in S_{\Theta }}\sum_{j\in S_{\Psi }}u_{a}^{l}(\theta_{i},\psi_{j})\cdot x_{i}\cdot q_{j}^{l},$$

In a one-shot game, when the chemical plants are expanded to many types, the payoff for the inspection agency is converted to Formula (12).

(12) $$u_{d}(x,q^{1},\ldots ,q^{N})=\sum_{l}p^{l}\cdot u_{d}^{l}(x,q^{l})=\sum_{l}p^{l}\cdot \sum_{i\in S_{\Theta }}\sum_{j\in S_{\Psi }}u_{d}^{l}(\theta_{i},\psi_{j})\cdot x_{i}\cdot q_{j}^{l}$$

where $q^{l}\;(l=1,\ldots ,N)$ where defines the probability distribution vector over the $l_{th}$ attacker’s strategy; and $p^{l}$ indicates the probability that the $l_{th}$ attacker occur.

Finally, based on Formulas (11) and (12), when a set of reasonable values for the parameters in Table 1 is determined, solutions can be computed through the baseline algorithms in Section 2.2.

#### 2.1.4. Solutions Concepts of the CPEP Game

Although the use of simultaneous games in the security domain is still common [19,29,30] in current game-theoretic modeling, three reasons proposed below make us prefer to model the CPEPs as sequential games.

Firstly, playing sequentially (i.e., chemical plants take their actions after observing the action taken by the inspection agency) better reflects the practice reality in a chemical industrial park. In this paper, it is often the case that the inspection agency commits to her strategy first, and then the chemical plants intelligently plan their infraction schedules after observation. That is to say, the chemical plants not only are able to collect information about the chemical industrial park, but they can also gather information about the inspection agency’s strategies. Therefore, it is reasonable to assume that the attackers have both complete and perfect information of a sequential game [31].

Secondly, playing sequentially can bring a higher payoff to the inspection agency. In SSGs, if the defender is permitted to implement her mixed strategy first, she will acquire the so-called “First-Mover Advantage” [32]. Moreover, Letchford [32] proved that the payoff of the defender from the mixed strategy is no less than that from simultaneous move. Based on the “First-Mover Advantage”, the inspection agency could choose to play a mixed strategy and then make her strategy public to enforce the game to be a sequential game which is beneficial to her.

Thirdly, playing sequentially can avoid the problem of equilibria selection. The Nash Equilibrium [33] is the most common solution concept obtained by computing the outcome in a simultaneous game. Since our CPEPs are not zero-sum games, it is highly possible to have multiple NE solutions [34] in practical case. Playing sequentially can make our CPEPs predictable and controllable for the inspection agency because the Strong Stackelberg Equilibrium (SSE) proposed by Leitmann [35] can ensure a unique solution in sequential games. Furthermore, Von Stengel and Zamir [36] introduced the ideal theory that the defender can choose the strategy which is close to the equilibrium solution, so that the attackers tend to choose a strategy which is beneficial to the defender, so as to achieve the SSE.

In addition, chemical plants can be expanded into many types because their main products are different, leading to different payoffs for the chemical plants and the inspection agency. In this situation, our CPEPs evolve into Bayesian Stackelberg Security Games which are the most common used games for reasoning about uncertainties while taking payoffs of attackers into account. Besides, the aim of the inspection agency is choosing a mixed strategy to maximize her payoff when best responses of all types of the chemical plants are considered. We name the best solution in this situation as the Bayesian Stackelberg Equilibrium (BSE) [37]. Therefore, the SSE solution and the BSE solution are defined as the solution concepts in this paper rather than the NE solution.

### 2.2. Baseline Algorithm to Solve the CPEP Game

There are two baseline algorithms utilized in this paper: the MultiLPs (Multiple Linear Programmings) algorithm and the DOBSS (Decomposed Optimal Bayesian Stackelberg Solver) algorithm. The MultiLPs algorithm was firstly proposed by Contizer and Sandhol [37], which is utilized to deal with CPEPs in the case that the game between the inspection agency and a certain type of chemical plant is computed. Interested readers are referred to Contizer and Sandhol [37].

As background information about CPEPs, the number of pure strategies for the attackers is growing exponentially as the types of attackers enlarge in the Harsanyi transformation [38] if the MultiLPs algorithm is used to solve the problem. In fact, the independence among the attackers could be modeled to design a new algorithm to solve this problem. DOBSS, currently the most efficient general Stackelberg solver [39], is applied for security scheduling at the Los Angeles International Airport which operates directly on the compact Bayesian representation. The key to the DOBSS decomposition is the observation that evaluating the defender strategy against a Harsanyi-transformed game matrix is equivalent to evaluating against each of the game matrices for the individual attacker types and then obtaining a weighted sum. Given prior probabilities $p^{l}$ for the chemical plants, the inspection agency solves the following problem formulation:

(13) $$\max_{x,q,a}\;\sum_{i\in S_{\Theta }}\sum_{l\in N}\sum_{j\in S_{\Psi }}p^{l}\cdot u_{d}^{l}(\theta_{i},\psi_{j})\cdot z_{ij}^{l},$$

(14) $$s.t.\;\sum_{i\in S_{\Theta }}\sum_{j\in S_{\Psi }}z_{ij}^{l}=1\;\forall l\in N,$$

(15) $$\sum_{j\in S_{\Psi }}z_{ij}^{l}\leq 1\;\forall l\in N,i\in S_{\Theta },$$

(16) $$q_{j}^{l}\leq \sum_{i\in S_{\Theta }}z_{ij}^{l}\leq 1\;\forall l\in N,j\in S_{\Psi },$$

(17) $$\sum_{j\in S_{\Psi }}q_{j}^{l}=1\;\forall l\in N,$$

(18) $$0\leq (a^{l}-\sum_{i\in S_{\Theta }}u_{a}^{l}(\theta_{i},\psi_{j})\cdot (\sum_{h\in S_{\Psi }}z_{ih}^{l}))\leq (1-q_{j}^{l})\cdot M\;\forall l\in N,j\in S_{\Psi },$$

(19) $$\sum_{j\in S_{\Psi }}z_{ij}^{l}=\sum_{j\in S_{\Psi }}z_{ij}^{1}=\sum_{j\in S_{\Psi }}z_{ij}^{2}=\ldots =\sum_{j\in S_{\Psi }}z_{ij}^{N}\;\forall l\in N,i\in S_{\Theta },$$

(20) $$z_{ij}^{l}\in [0,1]\;\forall l\in N,i\in S_{\Theta },j\in S_{\Psi },$$

(21) $$q_{j}^{l}\in \{0,1\}\;\forall l\in N,j\in S_{\Psi },$$

(22) $$a^{l}\in ℜ\;\forall l\in N$$

where M is a large positive number; the variable of $a^{l}$ is set to the maximum reward the $l_{th}$ attacker can receive, given the current policy of x taken by the defender; the notation of ${z^{l}}_{ij}$ represents ${z^{l}}_{ij}=x_{i}\cdot {q^{l}}_{j}$. The two inequalities in constraint five ensure that ${q^{l}}_{j}=1$ only for a strategy j that is optimal for follower type l. The constraint five can be explained in detail as follows: the leftmost inequality indicates that given the defender’s policy x, $a^{l}$ is the upper bound of the $l_{th}$ attacker’s utility for any strategy. While the rightmost inequality is inactive when ${q^{l}}_{j}=0$ because M is a large positive quantity. For the strategy that has ${q^{l}}_{j}=1$, the rightmost inequality can be transformed into $a^{l}\leq \sum_{i\in S_{\Theta }}\;u_{a}^{l}(\theta_{i},\psi_{j})\cdot x_{i}$, which incorporated with the leftmost inequality means this strategy must be optimal for the $l_{th}$ attacker.

### 2.3. Combined Study of the Game-Theoretic Model and the Source Estimation Methods

**Figure 2:**
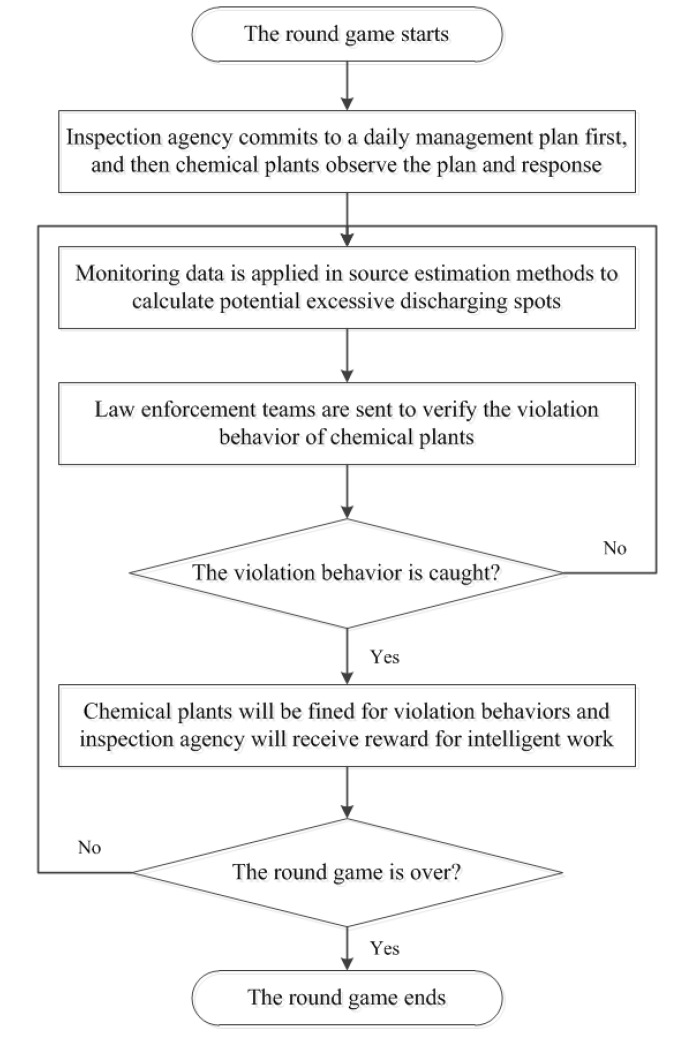
Workflow chart of combination work.

In this section, the combined study of the game-theoretic model and the source estimation methods will be further clarified. On the one hand, the developed game-theoretic model can generate executable daily management solutions of inspection resources. On the other hand, source estimation methods provide effective ways to detect the excessive discharging behaviors of chemical plants. Therefore, game-theoretic models in conjunction with source estimation methods can assist the decision-making process in daily management work and reduce the risks of atmospheric pollutants incidents. The combined study of the game-theoretic model and the source estimation methods, which is shown in Figure 2, follows the workflow below: (i) The inspection agency commits to a daily management plan first, subsequently the chemical plants can observe the plan and then take an action (i.e., discharge the excessive atmospheric pollutants or not) to optimize his reward; (ii) Real-time monitoring data collected by high-accuracy air quality monitoring stations and gas sensor modules serves as inputs for source estimation methods; (iii) After the potential releasing spots are calculated, the inspection agency would send a law enforcement team to check the situation; (iv) Once the infraction behaviors of chemical plants are confirmed, the chemical plants will be fined for a large amount of money. Moreover, the inspection agency would receive reward for this brilliant work; (v) Players repeat Steps i–iv until this game ends.

In conclusion, CPEPs constituted of players (i.e., the inspection agency and the chemical plants), strategies (i.e., actions taken by the inspection agency and the chemical plants respectively), payoffs (i.e., reward or penalty based on the corresponding action) and solution concept are firstly built up in this study through combining a game-theoretic model and source estimation methods. Then, the corresponding baseline solver-DOBSS is recommended to deal with CPEPs. Moreover, the combination work between the game-theoretic model and source estimation methods as well as workflow of real industrial practice is further extended. Finally, a workflow chart is presented to show how the proposed CPEPs work.

## 3. Case Study

In this section, an illustrative case study between the inspection agency and a certain type of chemical plant is conducted to show how the proposed CPEPs work. Besides, a practical case study conducted in Shanghai chemical cluster is also used to elaborate and explain how the CPEPs work in a real industrial scene. The illustrative case study is demonstrated in Section 3.1. The study area of the practical case is introduced in Section 3.2. Section 3.3 illustrates the experimental results by implementing the CPEPs. Important experimental findings are discussed in Section 4.

### 3.1. Illustrative Case Study

To verify the effectiveness of the proposed model in this paper, we firstly focus on the game between the inspection agency and a certain type of chemical plant in one day. Then, a set of reasonable values for the parameters shown in Table 4 is determined by experts from the inspection agency. The unit for monetary values in Table 4 is RMB (i.e., Chinese Yuan (¥)). Here, the probability of successful detection of the infraction behaviors through the two inspection resources is set at 0.5 while the probability of successful detection through gas sensor modules is set at 0.1. Based on historical discharging data, the value of T is set at two (see also Section 2.1.2). The payoff matrix calculated using Formulas (10) and (11) is shown in Table 5. In this table, the chemical plant is the row player while the inspection agency is the column player. Thus payoffs in this paper can be represented as $\left(u_{a},u_{d}\right)$. Then, the MultiLPs algorithm is used to solve the problem.

**Figure 3:**
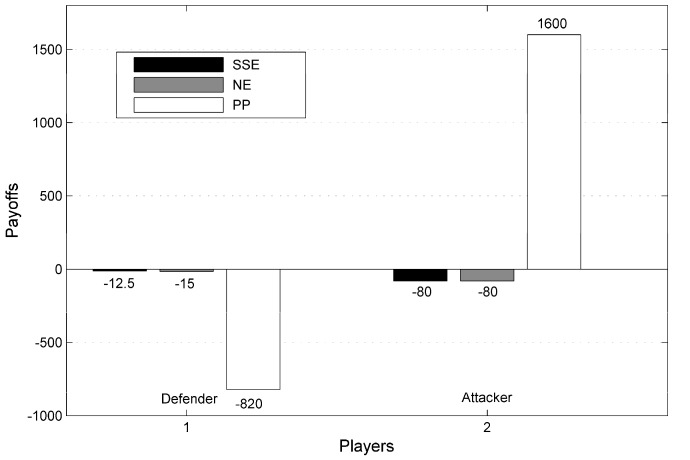
Payoffs for players in a one-day game under different solutions.

Based on the payoff matrix in Table 5 and enumerating pure strategies of the attacker, we solve four linear programs correspondingly. Then, the strategy profile is represented as $\left(q_{i};x\right)$ and results are illustrated as follows. Comparing the four solutions in corresponding linear programs, it can be concluded that the SSE solution is achieved at $\left(q_{4};x)=(1;0.2846,0.3404,0.3404,0.0346\right)$, in which the optimal payoffs are −12.5 and −80 for the inspection agency and the chemical plant respectively. In contrast, if the inspection agency would take the strategy which is denoted as $\theta_{1}$ in the past, then a payoff of −820 would be brought to her in one day because she did not have the ability to detect the irregularities of the chemical plants in the past inspection practice. Two Nash Equilibrium solutions are also acquired by dealing with corresponding solvers in our research, which are at $\left(q_{4};x)=(1;0.5567,0.1933,0.1933,0.0567\right)$ and $\left(q_{4};x)=(1;0.6157,0.1343,0.1343,0.1157\right)$ if both the inspection agency and the chemical plant choose to play simultaneously. Maximum payoffs for the inspection agency and chemical plant are −15 and −80, respectively, in the NE solution. Maximum payoffs for players in one day under different solution concepts are exhibited in Figure 3. The notation of PP in this figure is defined as payoff for the inspection agency, which is acquired from the present practice (i.e., operate the inspection resources all the time). By contrast, it reveals that on the one hand, the SSE solution satisfies the expectation of this paper for the inspection agency receiving more compliance and improving her payoff; on the other hand, the SSE solution is consistent with the assumption proposed in Section 2.1.4 that the chemical plant will choose the strategy of protecting the environment rather than ruining the environment when his optimal payoff is invariant under different pure strategies. Furthermore, it is obvious that the SSE solution outperforms the other solutions. Obviously, the illustrative case study indicates that daily management plans generated by the CPEPs outperform the previous industrial inspection practice taken by the inspection agency in assisting the decision-making process, controlling atmospheric pollution and reducing possibilities of leakage incidents or gaseous pollutants dispersion accidents.

From the illustrative case study, it is concluded that the problem is reasonable to be built into SSGs. By combining SSGs with source estimation methods, it not only solves the problem of detecting infraction behaviors of chemical plants, but also reduces operational costs for the inspection agency. In this way, this paper provides reasonable inspection plans for the inspection agency to supervise the production process of chemical industries intelligently. Furthermore, atmospheric pollutants’ abatement will contribute to improvement of the atmospheric environment.

### 3.2. Description of the Practical Case Study

**Figure 4:**
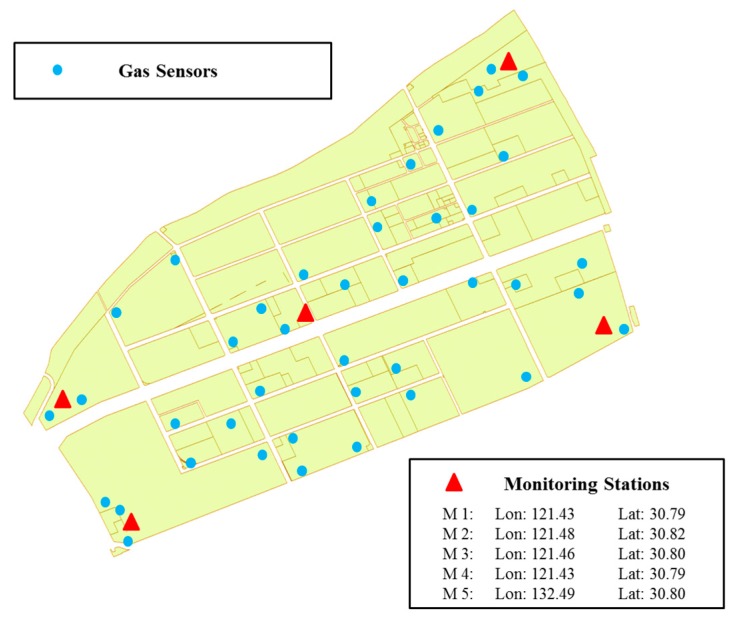
Layout of the case study.

**Figure 5:**
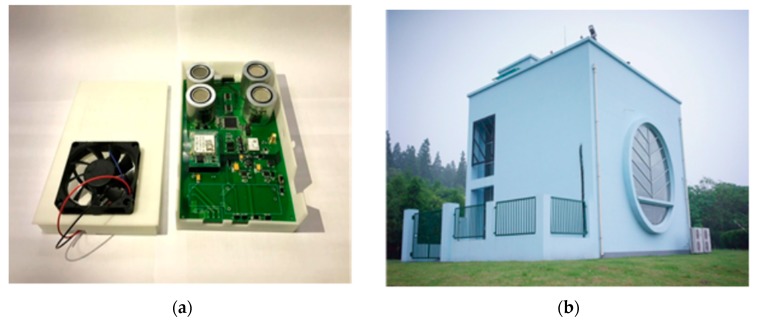
Inspection resources of inspection agency: (**a**) the gas sensor modules; and (**b**) one of the high-accuracy air quality monitoring stations.

Basically, a chemical industrial park is composed of numerous chemical companies, an inspection agency and functional departments (e.g., hospitals, hotels, police offices, etc.). Moreover, a chemical company may possess several chemical plants in the chemical industrial park. Our practical case is not an exception. Figure 4 shows a refinery from a chemical industrial park in Shanghai, China. The quadrilateral area (i.e., an approximately 2 km × 6.2 km area) is main region of the chemical industrial park. All the chemical plants are located in this district and all the inspection resources are also deployed in this area. The triangles indicate high-accuracy air quality monitoring stations, while the circles represent the gas sensor modules in Figure 4. Meanwhile, an illustrative picture of the two inspection resources is shown in Figure 5. The gas sensor modules [40] (i.e., the sensor probes are produced by Alphasense–The Sensor Technology Company, Essex, United Kingdom) utilized in this research are provided by SINGOAN Electronic Technology Co., Ltd. (Shenzhen, China) while the high-accuracy monitoring stations [28] are the product of Beijing Safety equipment manufacturing Co., Ltd., Beijing, China. In contrast, the measurement accuracy of high-accuracy air quality monitoring stations is usually a thousand times more accurate compared to that of gas sensor modules. These inspection resources (e.g., five monitoring stations and a total number of 310 gas sensor modules; each gas sensor module is placed in a 200 m × 200 m grid) are operating to inspect 55 chemical plants with 243 releasing spots. Moreover, specific information about the two inspection resources is listed in Table 6. It is worth noting that the high-accuracy monitoring stations as well as gas sensor modules are regularly calibrated by technical staff or automatic calibration devices to ensure the valid collections of monitoring data [41,42]. Indeed, a company in our case usually owns two or three chemical plants. These chemical plants produce similar products, and thus the byproducts generated during the production process are basically the same. Thus, two principles are proposed to classify the chemical plants sharing the same payoffs: (i) byproducts of these chemical plants are almost the same; and (ii) these chemical plants belong to the same company and locations of which are adjacent to each other. As a result, only 23 chemical plants with byproduct information are considered in our case, which is shown in Table A1. The occurring probabilities of chemical plants classified in one attacker’s type are accumulated as the occurring probability of the company. Furthermore, to meet the practical requirements in our modeling process, monitoring stations are open at the same time to collect data or shut down together to reduce costs because monitoring data utilized in source estimation methods are required to be diverse rather than data from only one monitoring station or two monitoring stations. Therefore, five monitoring stations are treated as one resource of inspection agency. Sample monitoring data collected by monitoring stations is listed in Table 7. The unit of these atmospheric pollutants is denoted as ${\mu g/m}^{3}$. One of the monitoring stations is named as Secco and loading time indicates the time when monitoring data is loaded into the database. Four main atmospheric pollutants, exhibited in Table 7, are SO_2_, H_2_S, NO and NH_3_.

Pure strategies for these chemical plants are the same as those in Table 2. Byproducts generated during the production process of the chemical plants are different as the company varies. Therefore, the payoffs for these chemical plants are changing accordingly depending on the characteristics of the byproducts. However, it is difficult to determine parameters of each attacker one by one owing to the large number of chemical plants. For the sake of simplicity, the lower bound and upper bound of some parameters (e.g., penalty for the defender and reward for the attacker) are determined by experts from the inspection agency. These parameters are assumed to obey a normal distribution between the intervals of the lower bound and upper bound. It is worth noting that if the models are implemented in industrial practice, all the parameters should be provided by experts. Here, it is worth noting that this information concerns estimations from the defender’s point of view. A series of parameters are given in Table 8. It can be found that the upper bound and lower bound of the penalty for the inspection agency are set at −350 RMB and −400 RMB, respectively, when she fails to catch the infraction behaviors of the chemical plants. Besides, the reward for the inspection agency catching the irregularities of the chemical plants is defined invariant because it accounts for a fixed proportion in the fining. In contrast, the penalty of the chemical plants is set to be invariant because of a fixed fine in the regulation. Furthermore, the upper bound and lower bound of reward for the chemical plants are set at 900 RMB and 800 RMB, respectively, in the case that he successfully discharges excessive atmospheric pollutants without purification treatment.

### 3.3. Experiments of the Practical Case Study

In this section, the perfect information (i.e., related parameters used to calculate payoffs) of the chemical plants, recognized by the inspection agency, is considered into experiments. Besides, the effect of detection probability on the results is also considered. Therefore, there are two experiments carried out: (i) a CPEP Game is conducted between the inspection agency and 23 chemical plants assuming that perfect information of chemical plants is determined; and (ii) an experiment to test how the value of $\gamma_{2}$ will impact the decisions of both players. The related parameters used to compute players’ payoffs are presented in Table A2.

Based on the previous monitoring data, the threat of different chemical plants can be calculated. It is assumed that the number of infraction behaviors conducted by the $l_{th}$ chemical plant in a year is $NUM^{l}$, which will be treated as the threat of the adversary. Thus the prior probabilities of these 23 types of chemical plants can be computed as $p^{l}=NUM^{l}\;/\;\sum_{l=1}NUM^{l}$. The corresponding prior probabilities with threats of these chemical plants are shown in Table A3. With the parameters and models above, the CPEP Game can be solved through the DOBSS.

#### 3.3.1. A One-Day Game with Perfect Information of Chemical Plants

In the case that the inspection agency has thorough information about the chemical plants and the chemical plants are able to observe the mixed strategy (i.e., daily management plan) adopted by the inspection agency, the BSE solution (i.e., probability distribution at different daily management plans) shown in Table 9 is computed through the DOBSS, and the corresponding maximum payoff for the inspection agency is −13.8.

Table 9 indicates that the inspection agency plays the strategy (open,open) at a probability of 0.38, plays the strategy (open,close) at a probability of 0.31, and so forth. In the BSE solution, all the chemical plants are compliant with the inspection agency by choosing the pure strategy of {no release,no release}. The detailed defender’s payoff with respect to different attacker strategies is exhibited in Table A4. The notations A_P and D_P in Table A4 represent the attacker’s payoff and the defender’s payoff, respectively. Besides, the notation of AP_one means the first pure strategy of the attacker, and so forth for the rest pure strategies. As shown in Table A4, it is worth noting that, if the chemical plants play strategies deviating from the BSE solution, the inspection agency would achieve a worse payoff. However, since the inspection agency knows exact information of the chemical plants, she is able to estimate the chemical plants’ best responses to her strategy and play accordingly. Meanwhile, it is also worth noting that chemical plants are believed to play their best response to maximize the inspection agency’s payoff in the assumption of SSE. For instance, the payoff of the fifteenth chemical plant is invariant to different pure strategies. However, if the fifteenth chemical plant chooses to play $\psi_{4}$, the inspection agency would acquire the highest payoff at −13.8 RMB. In the past inspection practice, the inspection agency kept the high-accuracy air quality monitoring stations and gas sensor modules on all the time, which means the defender is playing the strategy of {open,open}. However, almost none of irregularities conducted by the chemical plants can be detected by the inspection agency without source estimation methods. Therefore, in the past practice, the inspection agency acquired a payoff at −820 RMB and no compliance from the chemical plants. In contrast, it is obvious that the inspection agency improves her payoffs and acquires full compliance from the chemical plants when the CPEP game is played. Therefore, playing a CPEP game is essential for the inspection agency in her daily management work because the game not only reduces her daily operational costs and controlling the discharge behaviors of chemical plants, but also assists the daily decision-making process and reduces the possibilities of gaseous pollutants incidents.

#### 3.3.2. γ-Testing Experiment in One-Day Game

**Figure 6:**
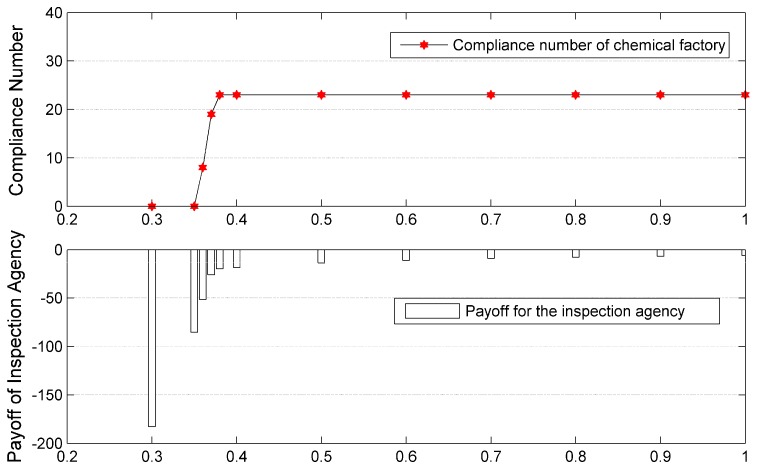
Number of chemical plants in compliance of environmental regulation and corresponding payoff of inspection agency (The abscissa is the variable value of $\gamma_{2}$).

In common sense, as the value of $\gamma_{2}$ increases (i.e., the prediction probability of infraction behavior is more accurate), the corresponding payoff of the inspection agency will be better. In this case, a $\gamma_{2}$-testing experiment is conducted to test how the value of $\gamma_{2}$ affects the payoff of the inspection agency and compliance of the chemical plants. In light of the fact that the value of $\gamma_{2}$ must be larger than the value of $\gamma_{1}$, the interval of $\gamma_{2}$ studied in this experiment is set between 0.3 and 1. Parameters in Table 8 which are used as inputs to test $\gamma_{2}$ stay invariant except the value of $\gamma_{2}$. Results in detail are shown in Table 10 and Figure 6. The notation of Def Strategy denotes the mixed strategy of the defender while the notation of Def Payoff means utility of the inspection agency under corresponding mixed strategy. Besides, the notation of Compliance Number indicates the number of chemical plants being compliant with the inspection agency. It can be derived from Table 10 that when the value of $\gamma_{2}$ is smaller than 0.35, the inspection agency would never acquire any compliance from the chemical plants although her mixed strategy converts to the pure strategy of opening the monitoring stations all the time. The critical value of $\gamma_{2}$ for the inspection agency to change her mixed strategy is 0.38. Meanwhile, all the chemical plants are compliant with the inspection agency when the value of $\gamma_{2}$ is higher than 0.38. Moreover, as the value of $\gamma_{2}$ increases, the opening duration of these monitoring stations reduces, and the corresponding payoff for inspection agency improves. The trend is clearly shown in Figure 6. The results indicate that it is essential to improve the predicting ability of source estimation methods if better management effectiveness on chemical plants is expected to be acquired.

## 4. Discussions

In this study, an illustrative case as well as a practical case was implemented to verify the effectiveness and practicability of CPEPs. Through the experimental results, two important findings could be observed. They are summarized as follows.

Our first finding is that the inspection agency is able to achieve compliance effectively from the chemical plants through CPEPs. Learning from the illustrative case and the first experiment in the practical case, all the chemical plants would be compliant in a NE solution, a SSE solution and a BSE solution when CPEPs are played. In these solutions, the inspection agency not only achieves a higher payoff, but also acquires more compliance than in its present practice. Moreover, more compliance from the chemical plants means that less atmospheric pollution would be discharged and the surrounding residential environment will be greatly improved correspondingly. Another finding is that the predicting ability of source estimation methods determines the performance of CPEPs. Learning from the second experiment in the practical case, it is concluded that as predicting ability of source estimation methods increases, the chemical plants are more likely to be compliant and the corresponding payoff for the inspection agency improves. Therefore, improving the predicting ability of source estimation methods in complicated surroundings becomes the focus of future research in this domain. 

However, there are some limitations in our results. Adversaries (i.e., the chemical plants in our study) are assumed to be fully rational in BSE solutions while they ought to be modeled according to bounded rationality in SSGs where attacks occur frequently. Due to a limited time for planning the attacks, attackers (i.e., the chemical plants) are often not the perfectly rational payoff maximizers. Thus, it is necessary to incorporate a behavior model of adversaries or robust optimization techniques with source estimation methods to deal with this difficult problem. Besides, the parameters related to the chemical plants are given by domain experts from the inspection agency, the exact value of which may be inaccurate. In the case that the inspection agency does not know the exact parameters of the chemical plants, she may assume that these parameters are located between certain minimal and maximal values (i.e., a parameter interval). In that case, a repeated game with the defender’s uncertainty on the attacker’s parameters should be played.

## 5. Conclusions

The aim of our research is to aid inspection agencies in effectively scheduling inspections of chemical production processes through providing executable daily management plans incorporating various real-world uncertainties and constraints. Previous work in this domain falls short of generating executable solutions for inspection agencies due to the following challenges: (i) inspection resources are limited and not fully utilized; (ii) source estimation methods are not applied in chemical industry management; and (iii) the number of adversaries is huge in this field. In addressing these challenges, this paper has advanced science with three originalities. The first originality is incorporating SSGs with source estimation methods intelligently. Second, two inspection resources (monitoring stations and gas sensor modules) are modeled into CPEPs. Third, simple data analysis on discharging information of adversaries is utilized to construct CPEPs.

Our experimental results show that the inspection agency will be able to achieve more compliance from the chemical plants and improve her payoff by playing CPEPs. It is worth noting that achieving more compliance from the chemical plants indicates less discharge of gaseous pollutants from chemical plants. Further, the surrounding ecosystem and residential environment will be largely improved on the one hand; the risks of hazardous gas leakage incidents or accidents will be considerably reduced on the other hand. It is also worth noting that improving payoff for the inspection agency means decreasing daily administrative expenses and at the same time improving profits. The two achievements meet the expectations of the environmental protection authorities.

Furthermore, our models and algorithms can be applied into real industrial practice. The practice follows the workflow below: (i) The inspection agency commits to a daily management plan first; subsequently, the chemical plants can observe the plan and then take an action (i.e., discharge the excessive atmospheric pollutants or not) to optimize their reward; (ii) Real-time monitoring data collected by high-accuracy air quality monitoring stations and gas sensor modules serve as input for source estimation methods; (iii) After the potential release spots are calculated, the inspection agency would send a law enforcement team to check the situation; (iv) Once the infraction behaviors of chemical plants are confirmed, the chemical plants will be fined for a large sum. Moreover, the inspection agency would receive reward for her work; (v) Players repeat Steps i–iv above until this game ends.

Future research on this subject is required to study the results of source estimation methods to model infraction behaviors of chemical plants, as well as consider models with bounded rationality of players.

## Figures and Tables

**Table 1:** Key notations used in this paper.

Notation	Explanation
N	Number of chemical plants
$\gamma_{1}$	Probability of detecting infraction behavior without opening monitoring stations
$\gamma_{2}$	Probability of detecting infraction behavior with opening monitoring stations
$C_{d}$	Operational costs of monitoring stations in the time unit for defender
$C_{a}$	Operational costs of Purification Treatment Plant in the time unit for attacker
$R_{l}^{a}$	Reward of the $l^{th}$ attacker discharging atmospheric pollutants but defender fails to detect the infraction behavior for attacker
$P_{l}^{d}$	Penalty of the $l^{th}$ attacker discharging atmospheric pollutants but defender fails to detect the infraction behavior for defender
$P_{l}^{a}$	Penalty of the $l^{th}$ attacker discharging atmospheric pollutants but defender successfully detects the infraction behavior for attacker
$R_{l}^{d}$	Reward of the $l^{th}$ attacker discharging atmospheric pollutants but defender successfully detects the infraction behavior for defender
$u_{d}$	Payoffs for defender in one game against N attackers
$u_{a}^{l}$	Payoffs for the $l^{th}$ attacker in one game against defender
$P^{l}$	Probability of the $l^{th}$ attacker occurrence
T	Time slices in a day

**Table 2:** Pure strategy of defender and attacker in one day with two time slices.

Notation	Defender’s Strategy	Notation	Attacker’s Strategy
$\theta_{1}$	{open,open}	$\psi_{1}$	{release,release}
$\theta_{2}$	{open,close}	$\psi_{2}$	{release,no release}
$\theta_{3}$	{close,open}	$\psi_{3}$	{no release,release}
$\theta_{4}$	{close,close}	$\psi_{4}$	{no release,no release}

**Table 3:** Payoff matrix in a time slice with only one defender and one attacker.

	Defender	Open	Close
Attacker			
**Release**		$(1-\gamma_{2})\cdot R_{l}^{a}+\gamma_{2}\cdot P_{l}^{a}$,$\gamma_{2}\cdot R_{l}^{d}+(1-\gamma_{2})\cdot P_{l}^{d}-C_{d}$	$(1-\gamma_{1})\cdot R_{l}^{a}+\gamma_{1}\cdot P_{l}^{a}$,$\gamma_{1}\cdot R_{l}^{d}+(1-\gamma_{1})\cdot P_{l}^{d}$
**No release**		$-C_{a}$,$-C_{d}$	$-C_{a}$,0

**Table 4:** Reasonable values of parameters.

Parameters	Value	Parameters	Value
$C_{d}$	10	$P_{l}^{a}$	−1600
$C_{a}$	40	$R_{l}^{d}$	600
$R_{l}^{a}$	800	$\gamma_{1}$	0.1
$P_{l}^{d}$	−400	$\gamma_{2}$	0.5
N	1	T	2
R	1		

**Table 5:** Payoff matrix of a one-day game between the defender and one attacker.

Strategy	$\theta_{1}$	$\theta_{2}$	$\theta_{3}$	$\theta_{4}$
$\psi_{1}$	−800, 180	160, −210	160, −210	1120, −600
$\psi_{2}$	−440, 80	−440, 90	520, −310	520, −300
$\psi_{3}$	−440, 80	520, −310	−440, 90	520, −300
$\psi_{4}$	−80, −20	−80, −10	−80, −10	−80, 0

**Table 6:** Specific information about the two inspection resources.

Inspection Resource	Monitoring Stations	Gas Sensor Module
Manufacturer	Beijing Safety equipment manufacturing Co., Ltd.	SINGOAN Electronic Technology Co., Ltd.
Quantity	5	310
Precision	1% of the measurements, usually 1 ppb	10% of the measurement range, usually 1 ppm

**Table 7:** Sample monitoring data collected by monitoring stations (${\mu g/m}^{3}$).

Monitoring Station	Loading Time	SO_2_	H_2_S	NO	NH_3_
**Secco**	26 July 2016 13:00:00	7.436	2.093	0.938	4.788
**Secco**	26 July 2016 12:55:00	7.436	2.254	1.072	5.548
**Secco**	26 July 2016 12:49:00	7.436	2.254	1.072	6.004
**Secco**	26 July 2016 12:45:00	7.436	2.254	1.072	6.004
**Secco**	26 July 2016 12:38:00	7.436	2.093	1.072	5.472
**Secco**	26 July 2016 12:35:00	7.436	2.254	0.938	6.926
**Secco**	26 July 2016 12:30:00	7.722	2.254	0.938	4.788

**Table 8:** Value of parameters.

Parameters	Value	Parameters	Value
$C_{d}$	10	$R_{l}^{a}\;\max$	900
$C_{a}$	40	$R_{l}^{a}\;\min$	800
$R_{l}^{d}$	600	$P_{l}^{a}$	−1600
$P_{l}^{d}\;\max$	−350	N	23
$P_{l}^{d}\;\min$	−400	$\gamma_{2}$	0.5
$\gamma_{1}$	0.1	T	2

**Table 9:** Defender’s Bayesian Stackelberg Equilibrium Strategy.

Strategy	Probability
(open,open)	0.38
(open,close)	0.31
(close,open)	0.31
(close,close)	0

**Table 10:** Results of one-day game when the value of $\gamma_{2}$ changes.

Value of $\gamma_{2}$	Def Strategy	Compliance Number	Def Payoff
0.3	(1, 0, 0, 0)	0	−182.7761
0.35	(1, 0, 0, 0)	0	−85.435
0.36	(1, 0, 0, 0)	8	−51.5572
0.37	(1, 0, 0, 0)	19	−25.9322
0.38	(0.9857, 0, 0, 0.0143)	23	−19.7143
0.4	(0.84, 0.08, 0.08, 0)	23	−18.4
0.5	(0.38, 0.31, 0.31, 0)	23	−13.8
0.6	(0.104,0.448,0.448, 0)	23	−11.04
0.7	(0, 0.46, 0.46, 0.08)	23	−9.2
0.8	(0, 0.3943, 0.3943, 0.2114)	23	−7.8857
0.9	(0, 0.345,0.345, 0.31)	23	−6.9
1.0	(0, 0.3067,0.3067, 0.3866)	23	−6.1333

## Appendix A.

**Table A1:** Main byproducts information of 23 chemical plants.

Chemical Plant	Pollutants Generated
*chemical plant a*	Particulates
*chemical plant b*	CO, SO_2_, NO_x_, HF, HCL
*chemical plant c*	SO_2_, NO_x_
*chemical plant d*	HCL, CH_3_CL, CH_3_COCH_3_, CH_2_CL_2_, C_6_H_6_, C_7_H_8_
*chemical plant e*	SO_2_, NO_x_, CO, VOC
*chemical plant f*	NO_x_, C_7_H_8_, CO, SO_2_, HCL, CL_2_
*chemical plant g*	HCL, C_7_H_8_, C_8_H_10_, C_6_H_5_CL, C_2_H_5_CL, SO_2_, NO_x_, VOC, HCL
*chemical plant h*	SO_2_, Particulates
*chemical plant i*	SO_2_, NO_x_, CO
*chemical plant j*	CO, SO_2_, NO_x_, CH_3_OH, CH_2_O
*chemical plant k*	SO_2_, NO_x_, Particulates
*chemical plant l*	NO_x_
*chemical plant m*	CO, SO_2_, NO_x_, HCL, CL_2_, Particulates
*chemical plant n*	CH_3_COCH_3_
*chemical plant o*	SO_2_, NO_x_, VOC
*chemical plant p*	NO_x_, VOC, NH_3_
*chemical plant q*	SO_2_, NO_X_, CO, HF, HCL, C_6_H_6_
*chemical plant r*	NO_x_, VOC
*chemical plant s*	C_6_H_7_N, SO_2_, NO_x_, HF, HCL
*chemical plant t*	COCL_2_, HCL, CO
*chemical plant u*	COCL_2_, HCL, CO SO_2_, NO_x_
*chemical plant v*	C_6_H_5_CL, CHCL_3_, C_2_H_5_CL, CCl_4_
*chemical plant w*	NH_3_, CO SO_2_, NO_x_

**Table A2:** Related parameters used in practical case study for solving CPEPs.

Chemical Plant	Penalty for Defender	Reward for Attacker
*chemical plant a*	−368	854
*chemical plant b*	−361	887
*chemical plant c*	−353	826
*chemical plant d*	−351	832
*chemical plant e*	−391	812
*chemical plant f*	−393	894
*chemical plant g*	−365	865
*chemical plant h*	−396	848
*chemical plant i*	−374	864
*chemical plant j*	−373	855
*chemical plant k*	−357	865
*chemical plant l*	−376	854
*chemical plant m*	−380	872
*chemical plant n*	−366	852
*chemical plant o*	−363	900
*chemical plant p*	−374	822
*chemical plant q*	−383	810
*chemical plant r*	−393	811
*chemical plant s*	−371	806
*chemical plant t*	−387	840
*chemical plant u*	−398	845
*chemical plant v*	−362	836
*chemical plant w*	−388	877

**Table A3:** Prior probabilities of different chemical plants.

Chemical Plant	Prior Probability	Infraction Number
*chemical plant a*	0.0503	106
*chemical plant b*	0.0645	136
*chemical plant c*	0.0323	68
*chemical plant d*	0.0517	109
*chemical plant e*	0.0342	72
*chemical plant f*	0.0517	109
*chemical plant g*	0.0527	111
*chemical plant h*	0.0243	51
*chemical plant i*	0.0327	69
*chemical plant j*	0.0313	66
*chemical plant k*	0.0517	109
*chemical plant l*	0.0598	126
*chemical plant m*	0.0371	78
*chemical plant n*	0.0565	119
*chemical plant o*	0.0517	109
*chemical plant p*	0.0214	45
*chemical plant q*	0.0484	102
*chemical plant r*	0.0389	82
*chemical plant s*	0.0626	132
*chemical plant t*	0.0214	45
*chemical plant u*	0.0422	89
*chemical plant v*	0.0404	85
*chemical plant w*	0.0422	89

**Table A4:** Defender’s payoff with respect to different attacker strategies.

Column Header	AP_One		AP_Two		AP_Three		AP_Four	
**Chemical plant**	A_P	D_P	A_P	D_P	A_P	D_P	A_P	D_P
*chemical plant a*	−137.4	−21.86	−108.7	−17.83	−108.7	−17.83	−80	−13.8
*chemical plant b*	−96.22	−13.13	−88.11	−13.46	−88.11	−13.46	−80	−13.8
*chemical plant c*	−172.4	−3.144	−126.2	−8.472	−126.2	−8.472	−80	−13.8
*chemical plant d*	−164.9	−0.648	−122.4	−7.224	−122.4	−7.224	−80	−13.8
*chemical plant e*	−189.8	−50.57	−134.9	−32.18	−134.9	−32.18	−80	−13.8
*chemical plant f*	−87.49	−53.06	−83.74	−33.43	−83.74	−33.43	−80	−13.8
*chemical plant g*	−123.7	−18.12	−101.8	−15.96	−101.8	−15.96	−80	−13.8
*chemical plant h*	−144.9	−56.81	−112.4	−35.30	−112.4	−35.30	−80	−13.8
*chemical plant i*	−124.9	−29.35	−102.5	−21.58	−102.5	−21.58	−80	−13.8
*chemical plant j*	−136.2	−28.10	−108.1	−20.95	−108.1	−20.95	−80	−13.8
*chemical plant k*	−123.7	−8.136	−101.8	−10.97	−101.8	−10.97	−80	−13.8
*chemical plant l*	−137.4	−31.85	−108.7	−22.82	−108.7	−22.82	−80	−13.8
*chemical plant m*	−114.9	−36.84	−97.47	−25.32	−97.47	−25.32	−80	−13.8
*chemical plant n*	−139.9	−19.37	−109.95	−16.58	−109.95	−16.58	−80	−13.8
*chemical plant o*	−80	−15.62	−80	−14.71	−80	−14.71	−80	−13.8
*chemical plant p*	−177.3	−29.35	−128.7	−21.58	−128.7	−21.58	−80	−13.8
*chemical plant q*	−192.3	−40.58	−136.2	−27.19	−136.2	−27.19	−80	−13.8
*chemical plant r*	−191.1	−53.06	−135.5	−33.43	−135.5	−33.43	−80	−13.8
*chemical plant s*	−197.3	−25.61	−138.7	−19.70	−138.7	−19.70	−80	−13.8
*chemical plant t*	−154.9	−45.58	−117.4	−29.69	−117.4	−29.69	−80	−13.8
*chemical plant u*	−148.6	−59.30	−114.3	−36.55	−114.3	−36.55	−80	−13.8
*chemical plant v*	−159.9	−14.38	−119.9	−14.09	−119.9	−14.09	−80	−13.8
*chemical plant w*	−108.7	−46.82	−94.35	−30.31	−94.35	−30.31	−80	−13.8
